# A Computational Model of Non-optimal Suspiciousness in the Minnesota Trust Game

**DOI:** 10.5334/cpsy.82

**Published:** 2022-04-06

**Authors:** Rebecca Kazinka, Iris Vilares, Angus W. MacDonald

**Affiliations:** 1 Graduate Program in Clinical Science and Psychopathology Research, University of Minnesota, Minneapolis, MN, US; 2 Psychology Department, University of Minnesota, Minneapolis, MN, US

**Keywords:** Spite sensitivity, trust, suspiciousness, decision-making, risk aversion, computational modeling, inequity aversion

## Abstract

This study modelled *spite sensitivity*, the worry that others are willing to incur a loss to hurt you, which is thought to undergird suspiciousness and persecutory ideation. Two samples performed a parametric, non-iterative trust game known as the Minnesota Trust Game (MTG). The MTG distinguishes suspicious decision-making from otherwise rational mistrust by incentivizing the player to trust in certain situations but not others. In Sample 1, 243 undergraduates who completed the MTG showed less trust as the amount of money they could lose increased. However, only for choices where partners had a financial *dis*incentive to betray the player was variation in the willingness to trust associated with suspicious beliefs. We modified the Fehr-Schmidt (1999) inequity aversion model, which compares unequal outcomes in social decision-making tasks, to include the *possibility for spite sensitivity*. An anticipated partner’s dislike of advantageous inequity (i.e., guilt) parameter included negative values, with negative guilt indicating *spite*. We hypothesized that the anticipated guilt parameter would be strongly related to suspicious beliefs. Our modification of the Fehr-Schmidt model improved estimation of MTG behavior. Furthermore, the estimation of partner’s spite-guilt was highly correlated with choices associated with beliefs in persecution. We replicated our findings in a second sample. This parameter was weakly correlated with a self-reported measure of persecutory ideation in Sample 2. The “Suspiciousness” condition, unique to the MTG, can be modeled to isolate spite sensitivity, suggesting differentiation from inequity aversion or risk aversion. The MTG offers promise for future studies to quantify persecutory beliefs in clinical populations.

## Introduction

Social interactions can be sorted broadly into 4 categories — cooperative, selfish, altruistic, and spiteful — based on the consequences (positive or negative) for the actor and the recipient (Gardner & West 2006). Cooperative mutual benefits, selfishness, and altruism have received a good deal of attention in recent decades. The final category is less commonly studied: spite occurs when the actor is willing to take a loss to ensure that a partner also loses. Initially, spite may seem irrational because it leads to a loss for an individual. However, spite may have evolved as a form of kin selection, in which spite toward an unrelated recipient may endure when the beneficiary is genetically related, in a similar manner as altruism (Gardner & West 2006; West & Gardner 2010). An example in nature can be seen in the sterile soldier caste in poly-embryonic parasitoid wasps. The wasp eggs divide asexually when they are laid on the eggs of moth caterpillars, and a small portion become the sterile soldier caste. To create the soldier caste is costly to the wasp, as it takes energy to create new life, yet they will not reproduce. Additionally, the soldier caste is costly to other wasp larvae because soldiers preferentially seek out and kill larvae that are less related to themselves (Giron et al., 2004). While this may first seem counterintuitive, this behavior frees up resources for their clone-mates, thus improving the chances of their kin surviving. For more examples in nature, see review by West & Gardner (West & Gardner 2010). Interestingly, the Social Value Orientation literature has observed behaviors that maximize inequity at the risk of a lower reward, referred to there as competitiveness (Murphy et al., 2011; Murphy et al., 2014). While we benefit from both literatures, the term spite may better encompass the threat felt by persecuted people. Here we leverage this conceptualization of spite to provide insight into one motivation for seemingly irrational negative outcomes in social interactions.

The existence of spite may provide clues for our understanding of a fear of spiteful partners, which we propose represents a fear of persecution. A fear of persecution, or increased suspiciousness that others are out to get oneself, is characteristic of psychotic disorders like schizophrenia (American Psychiatric Association 2013), but also exists within approximately 11% of non-clinical samples in the general population (Verdoux & Van Os 2002; Freeman et al., 2005; Bebbington et al., 2013). One possible mechanism for non-psychiatric suspiciousness of others may be this fear of their spiteful behavior. If an individual is socially alienated, they may be more inclined to interpret others’ behavior as spiteful. Therefore, strong priors that partners will be spiteful may provide a mechanism for persecution. Past research has suggested that increased self-reported suspiciousness is associated with *spite sensitivity*, or an individual’s fear that others are willing to incur a cost to themselves to cause the greater harm to the participant (Johnson et al., 2009; Wisner et al., 2021; Yu et al., 2021). This finding supports the framework that a fear of spite in social interactions may be key to understanding the fear of persecution seen both in the general population and in more serious clinical populations.

One approach to understanding an individual’s motivations in a social interaction is through game theory and social decision-making games. One such social decision-making game is the Trust Game (Berg et al., 1995), in which two players make decisions in succession about whether to cooperate or betray their partner. Player 1 (the investor) has the option to split an amount of money (e.g., $10) and give some portion (or all) to their partner player 2; it is then multiplied (usually by 3), given to player 2 (the trustee), and then player 2 can decide how much to return. The amount given by the investor is used as a proxy for the level of *trust* they have in the trustee, and the amount returned by the trustee is used to measure their level of *trustworthiness*. Individuals with psychosis, who commonly experience delusions of persecution (Applebaum et al., 1999), have been shown to trust less when playing as the investor (Fett et al., 2012; Fett et al., 2016). Thus, the Trust Game provides a useful foundation for understanding suspiciousness of partners, yet it is difficult to disentangle beliefs about the partner as suspiciousness, as rational mistrust of a predictably selfish partner, or as an aversion to uncertainty more generally.

**Figure 1:**
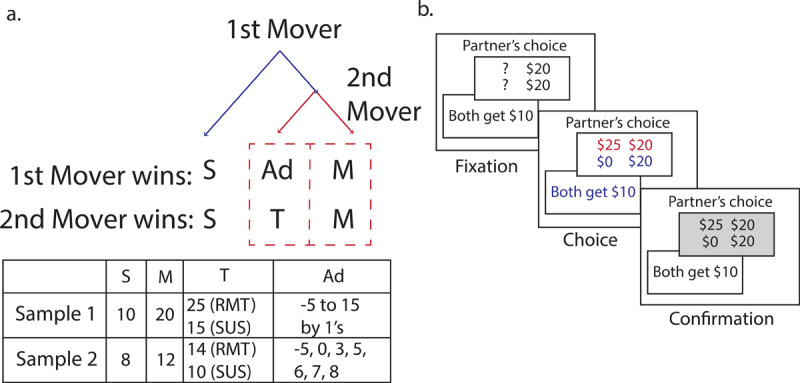
Diagram of gameplay. **a**. The Decision Tree of the Minnesota Trust Game shows possible outcomes for each player. The first mover first chooses between both players getting a small reward (S) or letting the second mover decide. The second mover can choose between both players getting a larger mutual reward (M) or an adverse payoff for the first mover (Ad) and a temptation T for the second mover. The table describes the values for S, M, T, and Ad for the two samples. The Suspiciousness condition is referred to as ‘SUS’ and the Rational Mistrust condition is referred to as ‘RMT’ when playing against a human partner. Participants also play against a fair coin, which are referred to as Risk aversion conditions or ‘RA(T)’, depending on the T amount. **b**. An example trial of the Rational Mistrust condition in the First Mover Game, with T = 25 and Ad = 0 (Sample 1). Participants saw question marks for the variable outcomes during the fixation, and then had a fixed amount of time to choose when the values were displayed. Their choice was confirmed during the confirmation window; however, they never received feedback about the choice made by the second mover.

In the typical Trust Game, it is reasonable that people are concerned that their partners are untrustworthy, given the competing gains for player 1 and player 2, and therefore players may be less willing to trust the partner. But what if player 2 incurs a cost in order for player 1 to lose money? In this instance, player 2 would be acting spitefully, and thus a non-optimal suspiciousness toward player 2 may indicate sensitivity to spite. As illustrated in ***Figure 1***, The Minnesota Trust Game (MTG) incorporates two conditions with outcomes dichotomized into two choices for each player. The first mover (player 1) can choose to take a small, safe outcome (*S*) and end the game (no trust) or trust the second mover (player 2) to choose between two possible divisions of money. In the *Rational Mistrust* condition, the second mover can either choose a fair, mutually beneficial outcome (*M*) larger than *S*, or an outcome that yields a temptingly higher payoff (*T*) for the second mover and adverse payoff (*Ad*) for first mover. As Ad decreases, the first mover’s shift from trusting to choosing the safe outcome reveals their level of *rational mistrust* in this condition. In a second condition, the *Suspiciousness* condition, the second mover is instead offered a temptation (*T*) that is less than the fair option, such that it is monetarily advantageous for the second mover to choose the fair option *M*. Therefore, it is reasonable to trust the second mover because they have an incentive to cooperate; the first mover may not trust in this condition if they have increased *suspiciousness* of the second mover (***Figure 1***). Participants also play this game against a fair coin to examine differences in trust depending on if the partner is another human or indifferent chance. This additional comparison disentangles the first mover’s beliefs about intention and risk aversion, thus the conditions against the coin are referred to as *Risk Aversion*. The added dimension of the *Suspiciousness* condition allows us to measure spite sensitivity, unlike the original Trust Game.

Findings from the Minnesota Trust Game from our lab showed that individuals were more trusting in the Suspiciousness condition compare to the Rational Mistrust condition when playing against a human partner versus a random coin (Johnson et al., 2009). However, there was variability in the amount that individuals were willing to trust in the Suspiciousness condition. Individuals who reported higher Alienation, a Multiphasic Personality Questionnaire (MPQ) (Patrick et al., 2002) subscale measuring beliefs like betrayal and isolation, were less trusting in the Suspiciousness condition. Additionally, this game showed differences in the Risk Aversion responses when comparing high and low Harm Avoidance, a MPQ subscale measuring risk aversion (Johnson et al., 2009). These results show evidence of individual differences in the MTG that could therefore be more precisely estimated using a computational model to measure spite sensitivity in this two-player game.

One approach to understanding the interaction of players in social decision-making games is to create a normative model of behavior to explain decision-making processes. Fehr & Schmidt (1999) first proposed a normative model of behavior in several social decision-making games that involved splitting money between individuals (Fehr & Schmidt 1999). This inequity aversion model uses two parameters to calculate utility of the options: a player’s guilt (β; dislike of advantageous inequality) and envy (α; dislike of disadvantageous inequality) by calculating the modifier of the difference between the outcomes for each player (Eq. 1). From the utility of options, this model can be used to describe behavior when an individual is maximizing the utility of an outcome, incorporating social utilities (based on parameters) along with monetary value. Importantly, this model adds a deeper understanding of individual differences, rather than simply relying on aggregate analysis of the game such as the Nash equilibrium (Nash 1950; Nash 1951). The Fehr-Schmidt model therefore establishes a simple explanation for choices to cooperate with a partner based on the dislike of inequity between the two players.

## The current study

In this study we developed a computational model of Minnesota Trust Game First Mover Game (1^st^M Game), with the aim of replicating observed MTG behavioral results and more accurately measuring spite sensitivity. Our model is adapted from the Fehr-Schmidt inequity aversion model (Fehr & Schmidt, 1999), which parameterizes play between two individuals who may receive unequal rewards. The Fehr-Schmidt model does not explain instances when individuals do not trust when it might be logical, from a monetary perspective, to assume the second mover will be trustworthy. Therefore, we tested several parameters to create the best fitting model for the MTG in two samples, one for model development and a second for replication and extension of our primary hypotheses. Below are the hypotheses we tested for our model, which were developed before testing. These hypotheses were not preregistered.

*H1. The Fehr-Schmidt model will outperform a random model, which assumes that an individual would have a 50% chance of choosing either option (go for the safe option or trust)*. This test was a validity check to ensure that the Fehr-Schmidt model was an appropriate base model guide in our modeling.

*H2. Behavior will differ between decision agents (the coin and human partner) and will therefore require separate parameters to explain participants’ efforts to account for their partners’ incentives*. Due to the difference in behavior between the two decision agents, we tested model fits that applied additional parameters for the human decision agent.

*H3. Risk aversion (R_i_) will partially but not fully explain the distinction between decision-agent conditions (comparing coin and human partner)*. Risk aversion was tested by modifying the difference between the first mover’s potential outcome against the safe assured payoff (*S*). We hypothesized a risk aversion parameter would be necessary for the model to explain overall risk aversion. However, risk aversion alone would not distinguish the behavioral differences seen between the Rational Mistrust and Suspiciousness conditions.

In addition to testing these hypotheses, we compared two different models to assess spite sensitivity by modeling an estimation of the partner’s choice:

*H4a. Model spite sensitivity by allowing the estimated second mover’s guilt to be negative*. We applied the Fehr-Schmidt model to the partner’s choice to measure the first mover’s estimation of the second mover’s guilt for getting more money. Importantly, we allowed the second mover’s guilt to become negative, which was not the case in the original Fehr-Schmidt model. Fehr & Schmidt reported that they constrained guilt to be positive to “rule out the existence of subjects who like to be better off than others” (p. 824, Fehr & Schmidt, 1999); they posited that while these people likely exist, in the context of the experiment it would have minimal impact on equilibrium behavior. Positive second mover inferred guilt means that the first mover believes their partner would be fairer; in contrast, negative guilt values would suggest that the first mover believes the partner would be spiteful (and actually be willing to incur a cost to inflict a higher cost on their partner). While in the original Trust Game, one would not anticipate that negative values would be common or relevant to the question at hand; in the MTG they would provide insight into sensitivity to spite specifically. We hypothesized that the estimated spite-guilt parameter, where positive values represent guilt and negative values represent spite, would therefore be most correlated with the Suspiciousness condition, where the utility of inequity for the second mover (i.e., earning more than the first mover) is pitted directly against earning more money.

*H4b. Model spite sensitivity separate from the estimated second mover’s guilt, such that guilt and spite are both non-negative parameters*. Like hypothesis 4a, we tested whether guilt and spite were separate measures, as compared to negative or positive guilt. Here, we created two parameters both constrained to be positive, and set the analysis so that when modeling the first mover’s estimation of the second mover’s value of their choices, guilt reduced the utility of the choice of having more than the first mover while spite increased its utility.

## Methods

### Participants

All subjects provided informed written consent through the University of Minnesota Internal Review Board (#0302S41721) as part of studies conducted in 2005–2008. Sample 1 consisted of 251 undergraduate psychology students (age mean = 19.9 (3.4), range 18–46 years; 62% female; 12.9 (SD = 1.3) years of education) who completed the Minnesota Trust Game (MTG) and a series of personality questionnaires in individual lab sessions. Participants received course extra-credit for their participation and all game payments were imaginary. Eight subjects were excluded for incomplete data on the task. We also analyzed a second sample of data that was previously reported (Johnson et al., 2009). Sample 2 consisted of 82 undergraduate psychology students who were tested in batches of 4–14. Sample 2 participants received extra-credit for participating in the experiment and were also paid cash based on two randomly selected trials with random pairings with other study participants. There was no deception around the incentive structure for participants in either sample, as they were aware prior to starting the game. Nine subjects were excluded due to poor task comprehension and a pattern of inconsistent responding (Johnson et al., 2009).

### Questionnaires

The personality inventory included items from the Multidimensional Personality Questionnaire Brief Form (MPQ-BF) (Patrick et al., 2002). MPQ-BF subscales included the Alienation subscale. The Alienation subscale quantifies suspiciousness in day-to-day life and contains items such as “some people are against me for no good reason.” Items were randomized throughout the questionnaire and scored on a 4-point Likert scale (1 = always true, 2 = mostly true, 3 = mostly false, 4 = always false). T-scores were calculated for the MPQ-BF as per Patrick and colleagues (2002) using software available at dionysus.psych.wisc.edu/arl/downloads.html. We selected this measure because our sample was not a clinical sample, and would show lower ranges of explicit persecutory ideation. The use of this scale is consistent with previous work in this area (Johnson et al., 2009). We split groups into high and low Alienation scores using the median as the cutoff value.

### Minnesota Trust Game

The Minnesota Trust Game (Johnson et al., 2009) is a computerized, parametric, non-iterative economic decision-making task comprised of two sub-games, the First Mover and Second Mover Games. We asked individuals to play against two different decision-agents: another participant in the research study and a fair “coin” which made 50/50 decisions. The experimenter emphasized that the decisions the participant made would determine their own and another player’s winnings in a randomly selected trial. In the case of a coin, the participant would flip the coin to determine the outcome for that trial.

Participants played both the First and Second Mover Games, playing the Second Mover Game (2^nd^M Game) first. Our modeling focused only on the First Mover Game (1^st^M Game). For the 1^st^M Game, they made the first decision in a two-player turn-based game. Here the participant decided whether to accept a smaller, safe reward *S* ($10 for Sample 1) or have their partner decide the outcome and potentially increase their mutual reward for that trial (*M*; $20 for Sample 1). As shown in the top node of the decision-tree (***Figure 1***), while playing the role of the first mover the participant chose between the assured payoff *S* and the alternative payoff (to trust the second mover). Participants in the 1^st^M Game were told whether the decision-agent was another participant in the study or a coinflip representing a passive partner. The alternative payoff consisted of two conditions, Rational Mistrust and Suspiciousness, which was distinguished by the value of the second mover’s potential winnings, the temptation *T*. The range of the adverse payoff *Ad* is described in ***Figure 1***. No-risk trials (*Ad* values above *S*) provided a validity probe to determine whether participants understood the experimental manipulations and allowed us to examine risk tolerance across a spectrum of choices.

The Rational Mistrust condition was the first mover’s decision to trust or not when the second mover had a monetary incentive to choose the temptation over the mutual reward (*T* = $25 in Sample 1), leaving the first mover with the adverse payoff *Ad*. The Suspiciousness condition was set up so that the second mover had a monetary *dis*incentive to select the temptation *T* ($15 in Sample 1). When the decision agent was a random coin, the Risk Aversion conditions paralleled the Rational Mistrust and Suspiciousness conditions, but they differed in that the first mover chose between the assured payoff and allowing the coin to determine their winnings. Thus, choosing the assured payoff in this game indicated a simple aversion to that risk. Values for *T, M*, and *S* can be found in ***Figure 1***. The 2^nd^M Game had the same contingencies but was from the perspective of the second mover.

### Design

For Sample 1, each participant made 42 unique decisions in the simpler, 2^nd^M Game (21 decisions in each condition, to test adverse payoffs for each value –$5 to $15) and then made 84 unique decisions in the 1^st^M Game (21 in each condition for both partner and coin decision agents). The 2^nd^M Game was performed before the 1^st^M Game, to ensure that participants understood what their partner had been told when making decisions. For Sample 2, each participant made 14 decisions in the 2^nd^M Game, and then made the same decisions in the 1^st^M Game with the two decision agents (coin and human partner), summing to 28 choices. Choices were presented in a random order. For each choice participants were shown the fixed values of the assured payoff and mutual reward alongside changing elements, i.e., the type of decision agent (partner or coin), the temptation, and the adverse payoff. To focus on participants’ prior beliefs about their potential partners (rather than learning), there was *no feedback* regarding the outcome of participants’ decisions until the end of the experiment. That is, each trial was treated as a *single interaction*.

### Analysis

The outcome variable was the participant’s choices during the MTG. In the 1^st^M Game, the choices were coded ‘0’ if the participant chose the assured payoff and ‘1’ if the participant ceded the choice to the decision-agent. Our analyses focus only on the 1^st^M Game responses. We ran a repeated measures logistic regression to predict the trust decision with Condition (Rational Mistrust or Suspiciousness) by Alienation score (High or Low) by Adverse Payoff (each value offered) by Decision Agent (coin or human). We included a random effects variable of participant to incorporate multiple observations per participant. The regression models can be found in the supplemental materials. Furthermore, to get a discrete measure for each individual, we leveraged the parametric manipulation of the adverse payoff to determine each participant’s change point from trusting to not trusting by fitting their choices using a Heaviside function, which relies on maximum likelihood estimation. We calculated thresholds for the 2^nd^M Game to compare 1^st^M and 2^nd^M Game behavior across individuals (Figure S1). We conducted Spearman correlations (denoted *r*_s_) between the parameter estimations and thresholds for each condition. Finally, we conducted one-tailed Spearman correlations between the estimated spite-guilt parameter and MPQ-Alienation, which we predicted would be negatively correlated; we also examined the relationship between General Risk Aversion parameter and MPQ-Harm Avoidance, which we anticipated would be positively correlated. Both of these hypotheses were predicted from previous research with the MTG (Johnson et al., 2009).

### Modeling

The aim of this analysis was to create a model that provided behavioral realism, i.e., a model that accurately described the behavioral phenomenon seen in the MTG, where individuals choose not to trust in an instance where it might be reasonable to do so (Suspiciousness condition). Additionally, we intended for the model to provide interpretable results when comparing individual differences in behavior. A final goal was that the model would identify individual differences in paranoid beliefs that could be quantified by personality measurements. Therefore, we focus parameters on representing behaviors related to suspiciousness.

We developed normative models in MATLAB (MATLAB R2018b) based on the Fehr & Schmidt (1999) inequity aversion model (Fehr & Schmidt, 1999) of two-player trust games to calculate utility (*U*) of a choice:


Eq. 1 \[
{U_1}\left(x \right) = {x_1} - {\alpha _{1}}max\left\{ {{x_2} - {x_1},\,\,0} \right\} - {\beta _{1}}max\left\{ {{x_1} - {x_2},\,0} \right\}
\]

where x is defined as the offer, and the subscripts are simplified to players 1 & 2 (first mover and second mover, respectively). This included two main parameters: envy (*α_1_*) and guilt (*β_1_*). Envy described the participant’s dislike of potential unfairness when their partner receives a larger payment. Guilt described the participant’s dislike of potential unfairness (hence in terms of unequal distribution of payoffs) when offered more money than their partner.

We tested several different parameter combinations (always using Sample 1), optimizing model fit according to Bayesian Information Criteria (BIC) (Schwarz 1978). The benefit of BIC is that it corrects for a higher number of parameters. Parameters were inferred by the model by fitting the choices using maximum likelihood estimation. We examined the extent to which the various tested parameters represented *spite sensitivity*, a phenomenon whereby players distrust a partner despite the partner’s cost for betraying the player (see supplemental materials for more details). Our best fitting model defined spite sensitivity using a modified guilt parameter. For the purposes of understanding the direction of spite sensitivity, we will refer to it as the *estimated spite-guilt* parameter, as low values represent spite sensitivity, while high values represent guilt. To test individual differences, each parameter was estimated for each individual participant.

Based on the Fehr-Schmidt model, the utility of the assured payoff *U_ASSURED_* was always *S*, because there was no inequality between the two players, and the value was constant. Similarly, the utility of the mutual payoff *U_MUTUAL_* was always *M*. Our main target was modeling the utility of the adverse payoff (*U_ADVERSE_(x_1_)*). In its simplest form, the adverse payoff was the value of the amount of money received by player *1*.

However, the decision is not simply among these three options, but rather between one assured option and one alternative option: to trust the partner. Therefore, we modeled the utility of trusting the partner (*U_TRUST_(x_1_)*) by weighting the partner’s options with probability (*p*) that the partner will choose the adverse payoff (Eq. 2). In the simplest forms of the model, *p* is defined as .5 to denote random choice between the adverse and mutual payoffs. This connotes the Risk Aversion conditions. In more complex forms (i.e., modeling the estimation of the partner’s choice), *p* is calculated based on the estimated parameters (see H4a).


Eq. 2 \[
{U_{TRUST}}\left({{x_1}} \right) = p\left({{U_{ADVERSE}}} \right) + \left({1 - p} \right)\left({{U_{MUTUAL}}} \right);
\]

Finally, to incorporate a measure of stability in decision making in the individual, we included a parameter for inverse temperature (*λ*), which represented the level of randomness in a decision. This means that the higher *λ*, the more consistent an individual is on their decision. We use a softmax equation to calculate the probability of a choice using *λ* (Eq. 3). The probability for each choice was then compared to the choices of the individual to calculate the negative log likelihood, which was then minimized to identify the best parameter estimates.


Eq. 3 \[
probability\left({TRUST} \right) = \frac{{{e^{\lambda *{U_{TRUST}}}}}}{{{e^{\lambda *{U_{SAFE}}}} + {e^{\lambda *{U_{TRUST}}}}}};
\]

This setup was the basic modeling procedure. Specific models for each hypothesis are laid out in the supplemental materials, and we describe the best model in the results.

### Stopping criteria

The model captures the distinctions between Rational Mistrust and Suspiciousness conditions, as well as distinction between the coin and human partners. To test the efficacy of the model in capturing these distinctions, we applied the parameter estimates collected for each individual to simulate choice outputs and compared the outcome to the original choices.

### Data Simulation

Data was simulated using the parameters extracted by the participants to attempt to recover behaviors seen in the participants using MATLAB.

### Data Availability Statement

As our sample was collected before data repositories were common, our original consent form did not account for this type of data sharing. Currently we are unable to make our data publicly available. However, our code is publicly available on the Open Science Foundation *https://osf.io/fhqj6/*.

## Results

### Behavioral results

**Figure 2:**
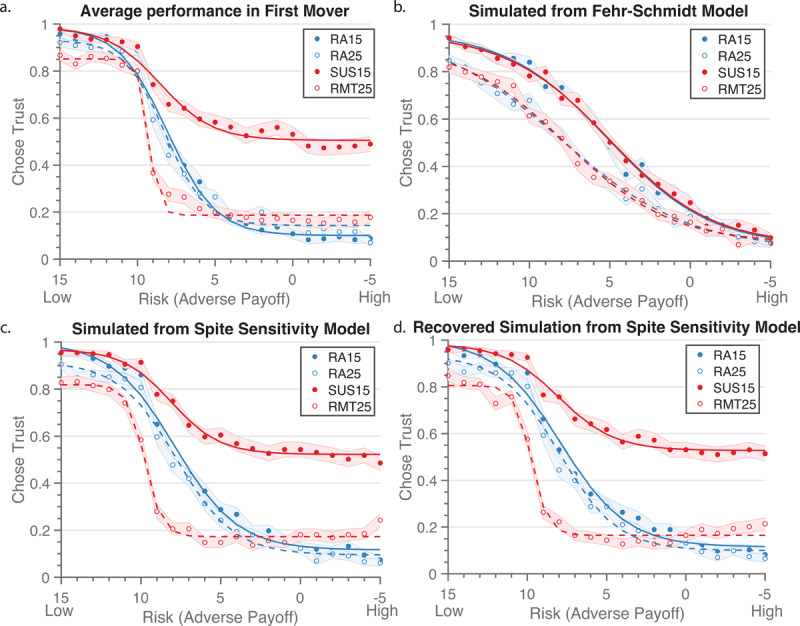
Behavior and Model Simulations. **a**) Original Sample 1 aggregate behavior. **b**) Simulated data from the original Fehr-Schmidt model, which does not match the pattern of Sample 1 aggregate behavior well. **c**) Simulated data from the best-fitting Spite Sensitivity model. **d**) We recovered parameters based on simulated data created in 2c and then re-simulated data based on the recovered parameters to test the stability of the model. Behavior is very similar to that seen in Figures 2a and 2c. Note, risk increases along the X-axis, as indicated by the decreasing Adverse Payoff. RA15 is the low temptation condition against the coin. RA25 is the high temptation condition with the coin. SUS15 is the Suspiciousness condition with the human partner. RMT25 is the Rational Mistrust condition with the human partner. The shading indicates 95% CI.

Looking at the behavior in the 1^st^M Game, we found that participants from Sample 1 showed similar behavior in the Risk Aversion conditions, yet less trust in the Rational Mistrust ($25) condition and greater trust in the Suspiciousness ($15) condition, although still well below 100%. A repeated measures logistic regression (temptation *×* decision agent *×* adverse payoff) identified a three-way interaction (Estimate = .002, SE = .0002, *t* = 10.2, *p* < .001). ***Figure 2a*** shows the interaction of temptation *×* decision agent (Estimate = –.037, SE = .001, *t* = –27.8, *p* < .001), in which there were differences in trust between the human partner conditions, but not coin conditions. This result confirms that participants treated the conditions for the human partners differently than the conditions with coin partners. Additionally, there were the predicted interactions of temptation *×* adverse payoff (Estimate = –.0004, SE = .00012, *t* = –3.63, *p* < .001) and adverse payoff x decision agent (Estimate = –.051, SE = .004, *t* = –14.7, *p* < .001). The relationship between temptation and adverse payoff showed that participants were more likely to trust in high-risk trials during the Suspiciousness condition compared to the Rational Mistrust condition. The adverse payoff x decision agent interaction showed that overall, participants were more trusting with the human conditions for lower values compared to the coin condition. We found main effects of temptation, decision agent, and adverse payoff (*p*’s < .01). These results held true when we controlled for age, sex, and education. Sex significantly predicted choice (Estimate = –.081, SE = .025, *t* = 3.31, *p* <.001), in which women were less trusting overall. Overall, these results replicated past findings in the Minnesota Trust Game (Johnson et al., 2009; Wisner et al., 2021; Yu et al., 2021).

### Model fitting

Our best-fitting model focused on behavioral realism to explain the spite sensitivity phenomenon using several parameters. Importantly, we set our goals to identify a model that had both a good fit (using Bayesian Criterion Information) and replication of the expected pattern of behavior seen in prior work, in which there was a significant increase in trust in the Suspiciousness condition only for the human partner. We used the Fehr-Schmidt inequity aversion parameter envy (*α*) for the first mover, and additionally set up the model to measure the first mover’s estimation of the second mover’s guilt (*β*′). Our model extends into negative values, where positive values represent a dislike of advantageous inequity (guilt), zero represents indifference, and negative values represent an enjoyment of advantageous inequity (spite). We refer to this parameter as *estimated spite-guilt* to represent the continuum. In addition, we included risk aversion parameters, which modulated the difference between the safe amount of money and the potential adverse payoff. One risk aversion parameter was set for all trials, while a second risk aversion parameter was added for only the human partner (which we refer to as social risk aversion). Finally, a softmax equation was used to estimate the inverse temperature (*i.e.*, noisiness) of decision making of the player, represented by lambda (*λ*). See methods section for more details.

### Model comparisons

To test models, we compared both BIC values and accuracy of simulated data in describing behavior (Table S1). The Fehr-Schmidt model (which included parameters for envy and inverse temperature) improved upon a random model (supplemental Figure S2); however, it does not separate out the interaction between condition and decision agent (***Figure 2b***). The Spite Sensitivity Model successfully reproduced the interaction of condition and decision agent; recovery of the model suggests good reproducibility (***Figure 2c*** & ***2d***). Correlations of original and recovered parameters can be found in Figure S3. Model comparisons tested risk aversion, estimated spite-guilt, separation of parameters across the decision agents, and spite as a separate parameter from guilt. Descriptions of model testing and results can be found in the supplemental materials. Importantly, estimated spite-guilt as a single continuous parameter performed better than separating estimated spite and estimated guilt, suggesting that H4a is better than H4b. The best fitting model had a lower BIC value than the average BIC for all tested models for 96% of participants, indicating that it was the best model for the majority of participants (Figure S4). Finally, we examined the extent that these variables were predicted by demographic variables such as age, education, and sex (Table S2).

**Figure 3:**
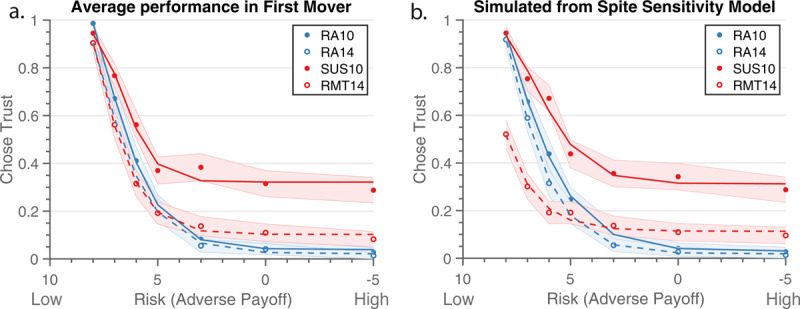
Replication of model comparisons. **a**) Average performance seen in Sample 2, in which participants in the Suspiciousness condition showed higher trust than the other conditions. **b**) While our model separates out the two partner conditions and in general replicated the original data well, it underestimated actual trust in the Rational Mistrust condition. Note, risk increases along the X-axis, as indicated by the decreasing Adverse Payoff. RA10 is the low temptation condition against the coin. RA14 is the high temptation condition with the coin. SUS10 is the Suspiciousness condition with the human partner. RMT14 is the Rational Mistrust condition with the human partner. The shading indicates 95% CI.

To test the replicability of the fit, we applied the Spite Sensitivity model to Sample 2, 73 participants from Johnson et al. (2009). The pattern of behavior in Sample 2 matched findings in Sample 1, in which the two Risk Aversion conditions were very similar, yet participants were less trusting in the Rational Mistrust condition, but more trusting in the Suspiciousness condition (***Figure 3a***). Specific behavioral results from this dataset can be found in Johnson et al. (2009). The simulated data from the Spite Sensitivity Model show the distinctions seen in the human partner between the Rational Mistrust and Suspiciousness conditions, yet little difference between the two Risk Aversion conditions. However, the simulated data of the Rational Mistrust condition underestimated the trust levels observed in the higher adverse payoff ranges (***Figure 3b***). Overall, the model adequately replicated the original behavior of the task, thus meeting our goal of generally fitting the pattern behavior seen in participants.

### Model parameters and individual behavior

**Figure 4:**
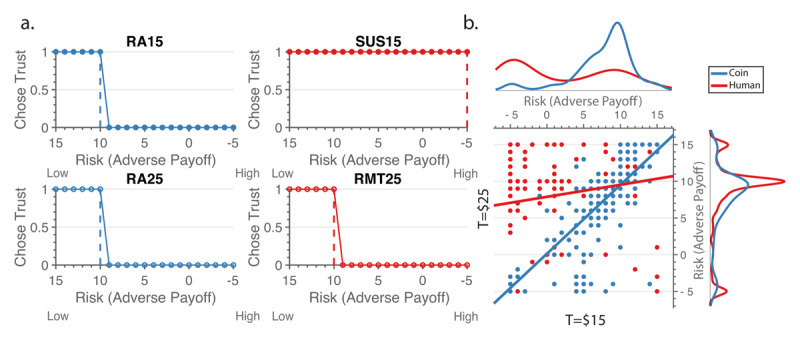
From individual choices to thresholds. **a**. Example individual results for behavior, showing Heaviside threshold (dotted vertical line) determined for condition. The y-axis is a binary choice between trusting (1) and not trusting (0). The x-axis shows the adverse payoff in reverse order, such that lower thresholds (further to the right) indicate increased risk. **b**. Scatter plot of Sample 1’s thresholds comparing across the low and high temptation T values for coin conditions (blue) and partner conditions (red). Beside each axis, histograms show the relative count for each condition. The change in temptation had little impact on individuals’ thresholds when the second mover was a coin but did when it was a human.

For Sample 1, we assessed individual differences by fitting a Heaviside threshold for each individual over each condition as a function of the adverse payoff value, which would allow for an approximation of the level of trust in each condition (see ***Figure 4a*** for sample participant). We successfully fit Heaviside thresholds to the individual data, where the fit predicted more than 94% of the decisions made on average. Thresholds that were lower indicated more willingness to incur risk, i.e., greater trust. Like previous findings, we found a high correlation between the Risk Aversion condition thresholds associated with both the coin partner conditions, suggesting equal concern with risk irrespective of temptation (*r*_s_ (241) = .784, p < .001; ***Figure 4b***), with the majority of thresholds at Ad = $10 (i.e., equal to the safe amount *S*). There was a lower correlation between the two partner conditions, in which the majority of thresholds of the Rational Mistrust condition were at $10 (*S*), but thresholds of the Suspiciousness condition were mostly at Ad = -$5 (*r*_s_ (241) = .175, *p* = .006). This lower correlation suggests that individuals treated the two conditions differently and that they are, in general, more willing to trust the partner when the partner is a human in the Suspiciousness condition.

To further assess how well the Spite Sensitivity model represented model-agnostic measures of behavior, we examined behavioral differences by comparing the thresholds with the estimated model parameters. The estimated spite-guilt parameter in Sample 1 most negatively correlated with the Suspiciousness condition thresholds (*r*_s_ (241) = –.38, *p* < .001). It also correlated negatively with the Rational Mistrust condition thresholds (*r*_s_ (241) = –.19, *p* = .003), but this correlation was smaller in magnitude (correlations significantly different, Meng’s Z = 2.38, *p* = .009). A negative correlation matched our expectations that as the estimated spite-guilt parameter decreases (suggesting more spite), the thresholds with the human partner would increase (suggesting less trust). Further, we expected the Suspiciousness condition to be most associated with the estimated spite-guilt parameter. Importantly, as participants do not receive any feedback during the game, this suggests that while some individuals believe their partner to be trustworthy, others do not trust the partner even in the Suspiciousness condition, where there is no evidence that they would be untrustworthy. The estimated spite-guilt parameter was not correlated with either Risk Aversion condition thresholds, which we anticipated as the estimated spite-guilt parameter was only applied to the human partner conditions.

The social risk aversion parameter was positively associated with the Rational Mistrust condition thresholds (*r*_s_ (241) = .25, *p* < .001); however, it was negatively correlated with the Suspiciousness condition thresholds (SUS15: *r*_s_ (241) = –.19, *p* = .004). This would suggest that in the Rational Mistrust condition, social risk aversion would increase as the risk of losing money increased, yet in the Suspiciousness condition social risk aversion would *decrease* as the risk increased. We did not anticipate this kind of relationship between social risk aversion and the Suspiciousness condition. Again, while there was a significant correlation between the social risk aversion parameter and the Suspiciousness condition, the correlation with the estimated spite-guilt parameter was higher (Meng’s Z = 5.26, *p* < .001). This relationship is examined further in the supplemental materials.

The general risk aversion parameter was highly correlated with all conditions’ thresholds, especially the coin conditions. However, it should be noted that the shape of the general risk aversion parameter was nonlinear such that the majority of individuals have a risk aversion near zero with a few outliers. This relationship may therefore represent a minority of participants and should be interpreted with caution. All other correlations comparing behavior and parameter estimates can be found in ***Table 1***, and selected correlations can be found in the supplemental materials (Figure S5). Correlations between the parameters for Sample 1 are shown in Figure S6.

**Table 1:** Correlations between Spite Sensitivity model parameters and MTG condition thresholds.


PARAMETER	RA-LOW	RA-HIGH	SUS-LOW	RMT-HIGH
		
		SAMPLE 1		
		
Inverse temperature	–.26***	–.23***	–.24***	–.11

Envy	.38***	.52***	.15*	.53***

Estimated spite-guilt	.02	.01	–.38***	–.19**

General Risk Aversion	.70***	.65***	.34***	.32***

Social Risk Aversion	.1	.13*	–.19**	.25***
		
		**SAMPLE 2**		
		
Inverse temperature	.06	–.13	.17	.11

Envy	–.11	–.15	–.36**	–.03

Estimated spite-guilt	–.11	–.05	–.53***	–.03

General Risk Aversion	.77***	.72***	.55***	.43***

Social Risk Aversion	–.13	–.15	–.15	.37**


*Note*: Spearman correlations for each parameter (rows) by condition (columns). RA-LOW is the Risk Aversion condition against the coin partner when temptation T was below the mutual payoff M; similarly, RA-HIGH refers to the Risk Aversion condition against the coin partner when temptation T was higher than the mutual payoff M. SUS-LOW refers to the Suspiciousness condition when temptation was below the mutual payoff for the human partner. RMT-HIGH refers to the Rational Mistrust condition when temptation was higher than the mutual payoff for the human partner. The top half of the table shows results for Sample 1, and the bottom half is for Sample 2. Italicized values represent results replicated in Sample 2. *p* < .05*, *p* < .01**, *p* < .001***.

We identified a computational model that replicated behavioral results in the MTG. In Sample 1, estimated spite-guilt was most strongly negatively associated with thresholds in the Suspiciousness condition, which has been associated with spite sensitivity behavior previously (Johnson et al., 2009). In Sample 2, we again found that the estimated spite-guilt parameter was most strongly negatively correlated with the thresholds in the Suspiciousness condition (*r*_s_(71) = –.53, *p* < .001), while it was not correlated with any other conditions, which we hypothesized. Like Sample 1, the estimated spite-guilt parameter was significantly more correlated with the Suspiciousness condition than the Rational Mistrust condition (Meng’s Z = 6.88, *p* < .001). When we look at the risk aversion parameters, we found that the general risk aversion was highly correlated with all conditions, as we saw in Sample 1. Finally, social risk aversion was only correlated with the Rational Mistrust condition thresholds (*r*_s_(71) = .37, *p* = .002), which was different from Sample 1 yet closer to our expectations. Like Sample 1, the social risk aversion parameter was more strongly correlated with the Rational Mistrust condition (Meng’s Z = 6.84, *p* < .001). Overall, these results validate our expectations that while both the estimated spite-guilt and the social risk aversion parameters only relate to the human partner, estimated spite-guilt is more strongly associated with the Suspiciousness condition, while social risk aversion is more associated with the Rational Mistrust condition. Again, there was a strong correlation between the social risk aversion parameter and the estimated spite-guilt parameter (*r*_s_(71) = .473, *p* < .001). Overall, these results from Sample 2 replicate findings seen in Sample 1. All correlations comparing behavior and parameter estimates can be found in ***Table 1***. Correlations between the parameters from Sample 2 can be found in the supplemental materials (Figure S7).

### Model parameters and personality measures

**Figure 5:**
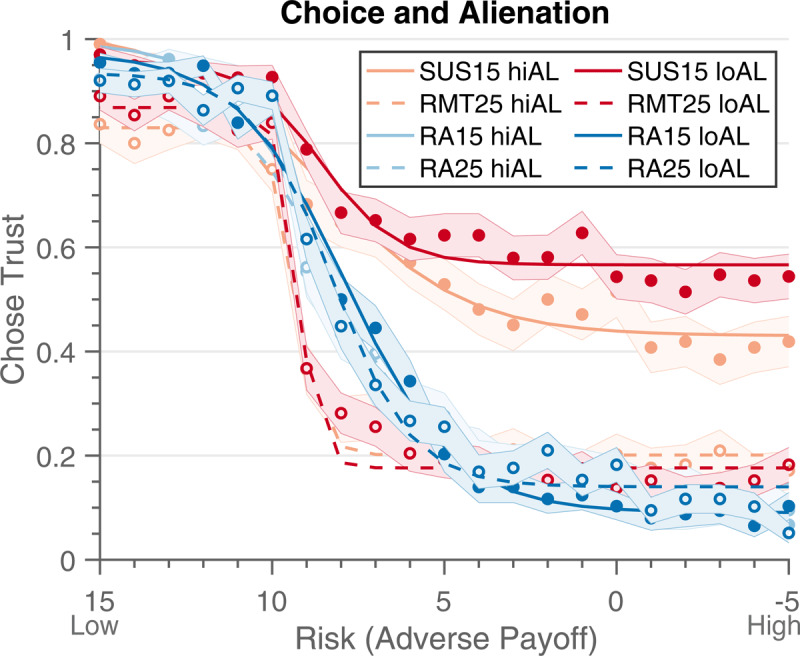
Sample 1 Behavior on First Mover game comparing Median Split of Alienation Scores. When comparing behavior in the first mover game between those with low (loAL) or high Alienation scores (hiAL), we found that those with higher Alienation scores are less trusting in the Suspiciousness condition (SUS15 loAL vs. SUS15 hiAL). There were not significant differences in between the other conditions when comparing high or low Alienation. Light and dark red lines are for conditions against the human partner, while light and dark blue lines represent conditions against the coin partner. Solid lines represent low temptation trials (T = 15), while dashed lines represent high temptation trials (T = 25). Light-colored lines represent high Alienation, while dark-colored lines represent low Alienation trials.

We additionally sought to compare individual differences on behavior and personality measures, namely the Multiphasic Personality Questionnaire (Patrick et al., 2002) subscales Alienation and Harm Avoidance, to the estimated parameters. We assessed the relationship of MPQ-Alienation, associated with persecutory ideations, to MTG behavior in Sample 1 to replicate past findings that high Alienation was associated with lower trust in the Suspiciousness condition (Johnson et al., 2009). We created median-split groups into high or low Alienation scores, and then conducted a repeated measures logistic regression (temptation × decision agent × adverse payoff × Alienation groups). We found the predicted four-way interaction effect (temptation x decision agent x adverse payoff x Alienation groups; Estimate = –.001, SE = .003, *t* = –2.93, *p* = .003; ***Figure 5***). As this is a small sample and four-way interactions are difficult to interpret, we examined additional interaction effects. Furthermore, there was an interaction of temptation × decision agent × Alienation (Estimate = .013, SE = .003, *t* = 4.65, *p* < .001). That is, when comparing the high and low Alienation groups there was a difference in thresholds that depended on the temptation and decision agent, such that those with higher Alienation were less trusting in the Suspiciousness condition but there was no difference in the other conditions. There was also an interaction of Alienation and decision agent (Estimate = –.301, SE = .055, *t* = –5.44, *p* < .001), which showed that those with higher Alienation were less trusting with human partners, but there was no difference between the two groups in terms of coin partners. There were no other significant interaction effects with Alienation (*p*’s >.4). When adding sex, education, and age, there were no major changes in these results; the interaction of temptation and adverse payoff was no longer significant (Estimate = –.0002, SE = .0001, *t* = 1.68, *p* = .092). This result suggests a meaningful relationship between behavior in the Suspiciousness condition and Alienation, such that those with higher Alienation are more likely to be less trusting specifically when dealing with the human partner in the Suspiciousness condition, as we have seen in previous studies.

We further examined the potential association between parameter estimates and personality measures from the MPQ. We were most interested in the Alienation subscale, which we anticipated would be related to estimated spite-guilt, and Harm Avoidance, which we anticipated would be associated with risk aversion (Figure S8). However, in Sample 1 we did not find a significant relationship between the general risk aversion parameter and MPQ-Harm Avoidance (*r*_s_(241) = .09, *p* = .090), nor estimated spite-guilt with MPQ-Alienation (*r*_s_(241) = –.05, *p* = .241). In Sample 2, there was also no correlation between general risk aversion and MPQ-Harm Avoidance (*r*_s_(71) = .06, *p* = .315), however there was the predicted correlation between estimated spite-guilt and MPQ-Alienation (*r*_s_(71) = –.21, *p* = .037). Sample 2 MPQ-Alienation and Harm Avoidance scores were within an expected range for a normative sample (MPQ-Alienation: mean = 25.0 (4.4), range = 15–37; MPQ-Harm Avoidance: 31.6 (5.0), range = 19–44). These results suggest there may be a connection between the estimate spite-guilt parameter and a self-report measure of alienation, a construct similar to suspiciousness.

## Discussion

This study’s aim was to create a computational model that accurately represented the patterns of behavior seen in the Minnesota Trust Game and provided clinically relevant parameters to represent persecutory beliefs. We created the model using a large sample of undergraduate students and then tested it on a second sample (Johnson et al., 2009). This study was the first attempt to build an econometric model of the unique circumstances examined in the Minnesota Trust Game. We developed and replicated in a separate sample a model based on the Fehr-Schmidt Inequity Aversion model that not only calculated players’ envy, but also their estimation of the second mover’s spite as an expansion of guilt into negative values. Our model performed better than both a random model and the original Fehr-Schmidt model (Fehr & Schmidt, 1999), which could not explain spite sensitivity in the MTG. The final model also included two risk aversion parameters, relating to overall risks and risks specific to the human interactions. The estimated spite-guilt parameter was most related to suspicious behavior in the task, suggesting that it captures spite sensitivity, whereas the general risk aversion was strongly associated with less trust in the game in all conditions. Social risk aversion, in contrast, was most strongly associated with Rational Mistrust. These results suggest that fear of spite and social risk aversion are separable mechanisms in decision-making. Furthermore, sample 2 replicated a significant relationship between MPQ-Alienation, a personality measure of persecution, and the Suspiciousness condition in the Minnesota Trust Game that specifically targets spite sensitivity.

In past studies of the Trust Game, participants tend to send less money when they know their partner is a computer (Johnson et al., 2011), suggesting they apply different expectations depending on the decision agent. In a similar two-person social decision making game, the Ultimatum Game, rejection of unfair options are more common for human than computer partners due to the expectation that humans, but not computers, would understand that it was unfair (Radke et al., 2012). In the Minnesota Trust Game, past research has found distinctions in behavior between the coin and human partners (Johnson et al., 2009). Taken together, these results show that the presumed intentions and mental capacity of a partner are critical to the social interaction. Our final model identified two instances in which treating the decision agents (coin and human) separately improved the model. Estimated spite-guilt (modeled as a prediction of the partner’s spite or guilt along a continuum) was only applied to the human partner. This parameter assumes some “mentalizing” on the part of the partner, but not coin; attempts to model mentalizing in the coin were not effective (see supplemental materials). Additionally, we had two separate risk aversion parameters for overall risk and risk associated with only the human partner interactions. While it is uncommon to separate risk aversion across two decision agents, it speaks to separate approaches to the two decision agents in our game. Due to the nature of our paradigm, treating the decision agents separately allowed us to measure distinct beliefs about the partners’ intentions.

Risk aversion is a common parameter used to assess the dislike of gambling or uncertainty in losing money (Levy 2010; Hula et al., 2018; Glimcher 2008; Hula et al., 2021). Past research points towards a difference between risk aversion and a lack of trust in the Trust Game (Bohnet & Zeckhauser 2004, Houser et al., 2010). Computational modeling of the multi-round Trust Game has shown that risk aversion (the value of money kept over potential money gained), played an important role comparing the potential loss regardless of the partner’s cooperativity (Hula et al., 2018). A recent study identified demographic differences in this model, particularly showing that males were less risk averse, consistent with our supplemental findings (Hula et al., 2021). Because we manipulated risk as a parametric variable, we anticipated that risk aversion would play an important role in decision making in this game yet would not fully explain behavior in the Suspiciousness condition. Our best model included risk aversion parameters with two different functions, as mentioned above. This social risk aversion parameter was also most associated with the Rational Mistrust condition, showing its ties to human mistrust. Against expectations, we did not find a significant relationship between MPQ-Harm Avoidance and general risk aversion (Johnson et al., 2009), perhaps because those questions focus on physical rather than financial risks. Overall, the inclusion of risk aversion parameters is valuable in contextualizing the fear of losing money versus partner distrust.

We found that the estimated spite-guilt parameter was important to model suspicious behavior, thus allowing us to model spite sensitivity for the first time (H4a). In contrast, separating positive and negative guilt into guilt and spite did not improve the model (H4b). Uniquely, this model extended the estimated spite-guilt parameter to include negative values. The original Fehr-Schmidt model did not allow for negative parameters in order to test specific individuals who deviated from equilibrium, and it is uncommon in other uses of this model (Yang et al., 2016). However, as we are interested in an individual’s perception of spiteful partners, including a negative range for estimated spite-guilt allowed us to do this parsimoniously as a continuous variable. We found one article that referred to spite and guilt as opposite responses in a public goods game (Chan et al., 1996). Guilt is thought to be an important driver in enhancing relationships and social norms (Baumeister et al., 1994). Modeling of the second mover’s perspective therefore focuses on guilt aversion, which encompasses both maximizing outcomes and minimizing anticipated guilt from letting down a partner (Battigalli & Dufwenberg 2006). However, guilt aversion is similarly only allowed to be positive, and testing the propensity for spite is less common in trust games; thus, we are limited in our ability to examine if spite and guilt are truly a continuum. Our results suggest it is an effective way to model fear of spite in the Minnesota Trust Game. We hope that future work can continue to assess the continuity of this measure, particularly as it might apply to more extreme ends of the spectrum (i.e., paranoid delusions or gullibility) in clinical populations. Importantly, as predicted this parameter was most associated with the Suspiciousness condition in both samples. The estimated spite-guilt parameter was key to understanding spite sensitive beliefs about a partner, above-and-beyond risk aversion.

While estimated spite-guilt captures the participant’s beliefs about a partner’s intentions, how well does it capture theory of mind or the capacity to represent one’s own and other persons’ mental states (Premack & Woodruff 1978)? As persecutory ideations are unfounded beliefs that another has ill intentions towards oneself, one theory is that they are driven by the inability to understand or intuit another’s intentions. Broadly, substantial theory of mind deficits have been observed in individuals with first episode psychosis, ultra-high risk for psychosis, and unaffected relatives (Bora & Pantelis 2013). Additional research suggests that these findings persist into chronic psychosis (Bora et al., 2009). However, the specificity of the role of theory of mind in persecutory delusions is less certain. Recent work has instead suggested that hyper-mentalization (i.e. over-attribution of intentions to random movements) in patients was associated with positive symptoms like delusions, namely suspiciousness, thus showing that persecutory beliefs may be related to poor theory of mind due to over – not under — use (Hajdúk et al., 2018). However, a recent meta-analysis of delusions suggested that the theory of mind account does not have strong support, instead finding theory of mind is more associated with negative symptoms (Garety & Freeman 2013) like flat affect, anhedonia, and apathy (Andreasen 1990). For the current study, we did not assess theory of mind deficits, and can only identify that participants believe that the human partner has distinct intentions from the coin, as they treated the two decision agents differently. It becomes more difficult to assess the role of theory of mind in decision making in the MTG across the conditions, as a failure to understand the motivation for the partner in the Suspiciousness condition may also look like distrust of the partner. Therefore, theory of mind may be a factor that we cannot directly address in the current study.

Other computational models exist that focus on understanding persecutory beliefs. The Social Inference model examined an advice-taking task, in which participants had to infer the accuracy and intention of the advice on a probabilistic lottery game, which was manipulated over time (volatility) and used these values to examine belief precision. Individuals with greater persecutory delusions were less likely to take advice and more likely to believe their advisor was intentionally providing false information (Wellstein et al., 2020). Hierarchical Bayesian modeling found that individuals with higher persecutory delusions are less likely to incorporate new contextual information into belief updating (Diaconescu et al., 2020). These results are similar to hierarchical Bayesian modeling applied to a moral inference task in patients with Borderline Personality Disorder (Siegel et al., 2020). Finally, the same modeling has been applied to a non-social probabilistic game and similarly suggested decreased belief-updating in individuals with greater persecutory beliefs (Reed et al., 2020). These theories are valuable in understanding the failed response to new information about a potential partner. Our emphasis on measuring the specificity of the circumstances in which a participants’ prior beliefs are differentially triggered based on selfish or spiteful motives is supported by these studies showing that a good deal of the signal associated with persecution lies in participants’ fixed prior beliefs.

There remain open questions for our model. There was some overlap in the relationships of the estimated spite-guilt and social risk aversion parameters. This included having both correlate with both the Suspiciousness and Rational Mistrust conditions, albeit with stronger relationships with the expected conditions. Additionally, social risk aversion and estimated spite-guilt parameters are correlated themselves. As both parameters were included in only the human partner trials, it is reasonable that both would be associated with the two human partner trials. Importantly, we were still able to recover the parameters with good replication of the expected outcomes in behavior despite these overlapping parameters. However, we did not predict a negative relationship between social risk aversion and the Suspiciousness condition thresholds. Additionally, we found that when there is a larger discrepancy between the two parameters, social risk aversion accounted for the lower trust in the Rational Mistrust condition and therefore had a negative correlation with the greater trust in the Suspiciousness condition. These findings suggest that social risk aversion and estimated spite-guilt parameters interact. One explanation for this relationship is that those who are specifically suspicious of their partner’s intent would also be more concerned about risk taking with a human partner in any situation. However, the estimated spite-guilt parameter is most strongly associated with the Suspiciousness condition in both samples.

We also found mixed results about the relationship between estimated spite-guilt and the MPQ Alienation scale, which we had hypothesized would be related. Both behavioral samples found that higher Alienation was associated with less trust in the Suspiciousness condition behavior when we separated the sample into high and low Alienation groups. However, there was only a significant correlation between Alienation and estimated spite-guilt in Sample 2. One explanation for the difference across samples may be that the two games varied in the values used for the game. It is also possible that the positive correlation between the self-reported measure of alienation and the estimated spite-guilt parameter was due to chance, as the sample size is lower on Sample 2. Alternatively, a potentially important difference between the samples is that we paid participants in the Sample 2, but not Sample 1, which may mean individuals were more incentivized to reveal their true preferences in Sample 2. There is mixed evidence that incentive payments influence decisions to trust differently than hypothetical payments. Previous research has shown a minimal difference in behavior between hypothetical and incentivized outcomes in the Trust Game (Thielmann et al., 2016). In a similar case, trust outcomes were similar when comparing monetary and non-monetary incentives (Luccasen 2014). However, the opposite has also been observed. A meta-analysis found that when making similar comparisons of real payments versus randomized lottery payments (i.e. randomly selecting an individual to actually pay them by their choice), individuals sent less money when it was a randomized choice (Johnson et al., 2011). Similarly, Holm & Nystedt (2008) found that those given hypothetical outcomes were much less willing to trust than those with real payments or random lottery payments (Holm 2008). In comparison with our results, it may be the case that real payment led to a difference in behavior in the task in Sample 2 compared to Sample 1, although it is difficult to assess with the current data. Finally, we also used undergraduate students for both samples, who may feel less persecuted than the broader community, thus reducing variability. This population is also not representative of the wider population, and therefore these results should be replicated in a population with a greater range of demographic characteristics. Future studies should disentangle between these different possibilities.

Our model of the Minnesota Trust Game is the first to distinguish behavior related to spite sensitivity that predicts behavior in the Suspiciousness condition, which assesses irrational fear of a partner’s likelihood to be spiteful. As spite is hypothesized to represent a behavior that is evolutionarily adaptive for kin selection (Gardner & West 2006), spite sensitivity may represent a prior expectation that out-group individuals will exclude or persecute an individual despite no direct benefit to the persecutor. This model is a first step to measuring persecutory ideation in a healthy sample in hopes of further elucidating mechanisms of persecutory ideation. Our findings support hypotheses that prior beliefs about a partner’s intentions influence decisions to trust. It will be useful to compare behavior in the task with individuals who have experienced persecutory ideation, and if successful to use it as a tool to examine the representation of spite sensitivity in the brain. Finally, modeling the Minnesota Trust Game could prove useful for examining treatments that target persecutory beliefs – for example, by allowing observations of an effect of these interventions on the estimated spite-guilt parameter.

## Additional File

The additional file for this article can be found as follows:

10.5334/cpsy.82.s1 Supplemental Materials. Includes additional hypothesis testing, demographic predictors, and Second Mover results.
