# Supplementary material for: A Computational Model of Non-optimal Suspiciousness in the Minnesota Trust Game
Source: Comput Psychiatr. 2022 Apr 6;6(1):60–78. doi: 10.5334/cpsy.82 (PMC11104362; doi:10.5334/cpsy.82)
Supplement: Supplemental Materials. — Includes additional hypothesis testing, demographic predictors, and Second Mover results. [file cpsy-6-1-82-s1.pdf]

Supplemental Materials

A Computational Model of Non-optimal Suspiciousness in the Minnesota Trust Game

Rebecca Kazinka[1], Iris Vilares[2], Angus W. MacDonald III[2]

1. Graduate Program in Clinical Science and Psychopathology Research, University of Minnesota, Minneapolis, MN, United States of America

2. Psychology Department, University of Minnesota, Minneapolis, MN, United States of America

Corresponding author: angus@umn.edu

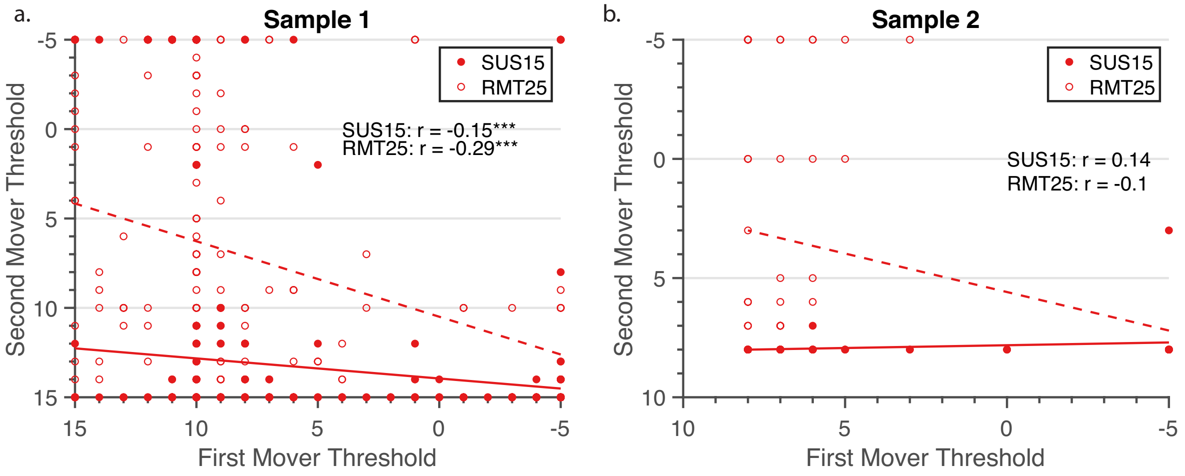

Figure S1. Correlations of First Mover and Second Mover Thresholds. *Spearman correlations of Sample 1 and b) Sample 2 showed that participants did not simply assume their partner was like themselves, but instead had a significant negative relationship in Sample 1 and a negative trend in Sample 2. This result suggests that those who were the least trusting in the First Mover Game were the most selfish in the Second Mover Game.* $p < .05$*, $p < .01$**, $p < .001$***

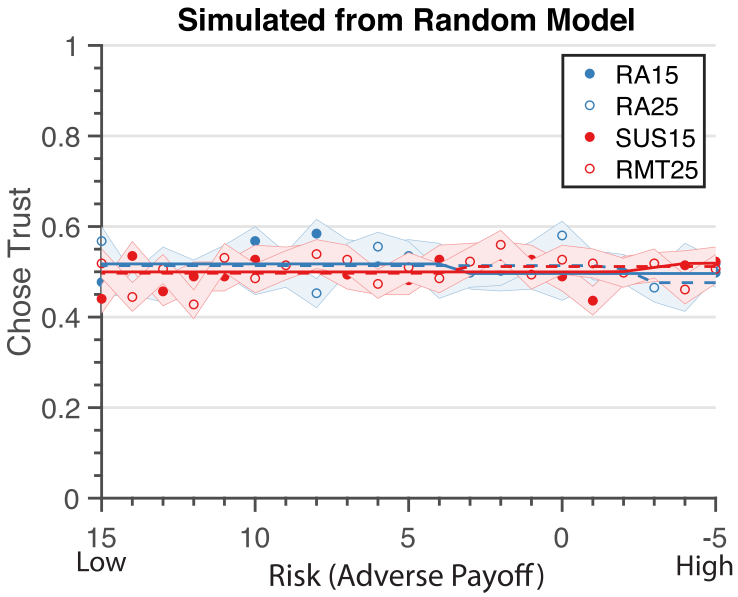

Figure S2. Random Simulated Model. *This model simulated responses in the game if they were assumed to be random. We see that, overall, all choices had a mean of .5 (as expected from our random model). Note, risk increases along the x-axis, as indicated by the decreasing Adverse Payoff. RA15 is the low temptation condition against the coin. RA25 is the high temptation condition with the coin. SUS15 is the Suspiciousness condition with the human partner. RMT25 is the Rational Mistrust condition with the human partner. The shading indicates 95% CI.*

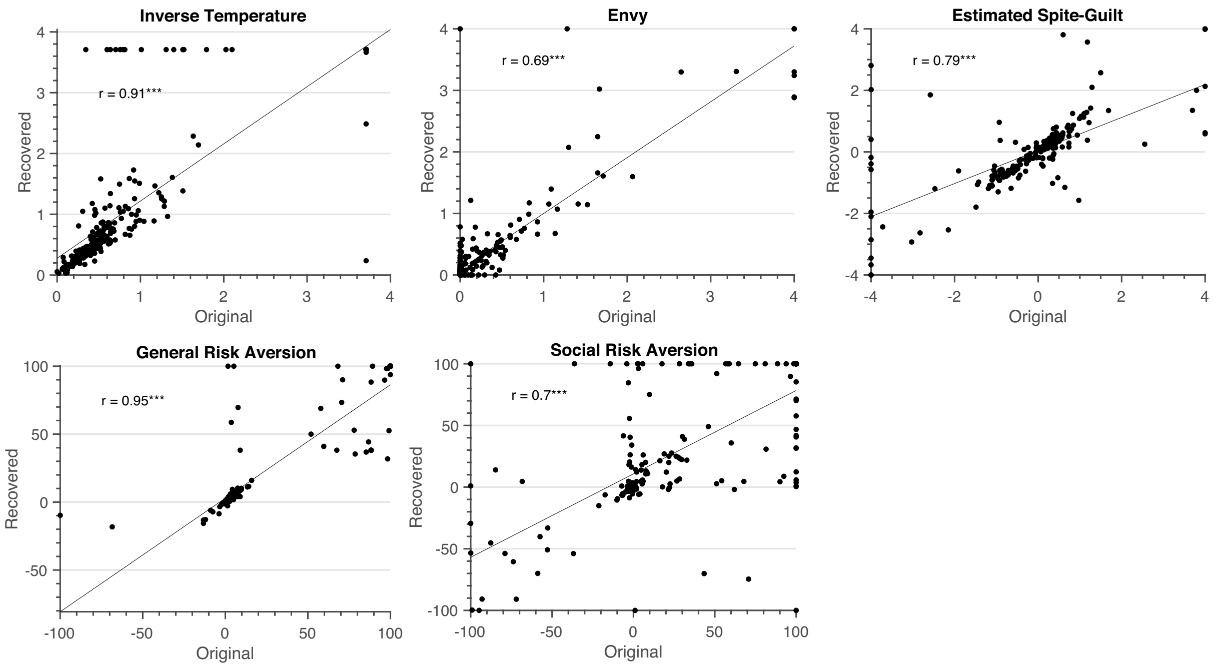

Figure S3. Correlations of original and recovered parameters. *All parameters showed good to excellent recovery. Correlations are Spearman. p < .05*, p < .01**, p < .001****

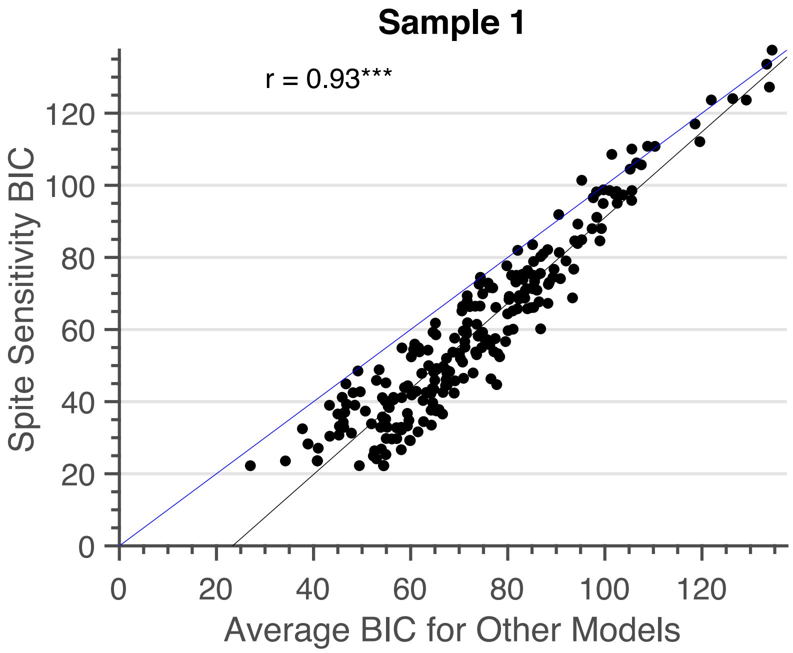

Figure S4. Comparison of best-fitting model BIC values to the average BIC of all other tested models. *The best-fitting model had a superior BIC value for 96% of participants. Blue line represents a theoretical perfect correlation with the Spite Sensitivity Model BIC values, showing that the other model BIC values fall above the Spite Sensitivity Model BIC values. However, these BIC values were still highly correlated.* $p < .05$*, $p < .01$**, $p < .001$***

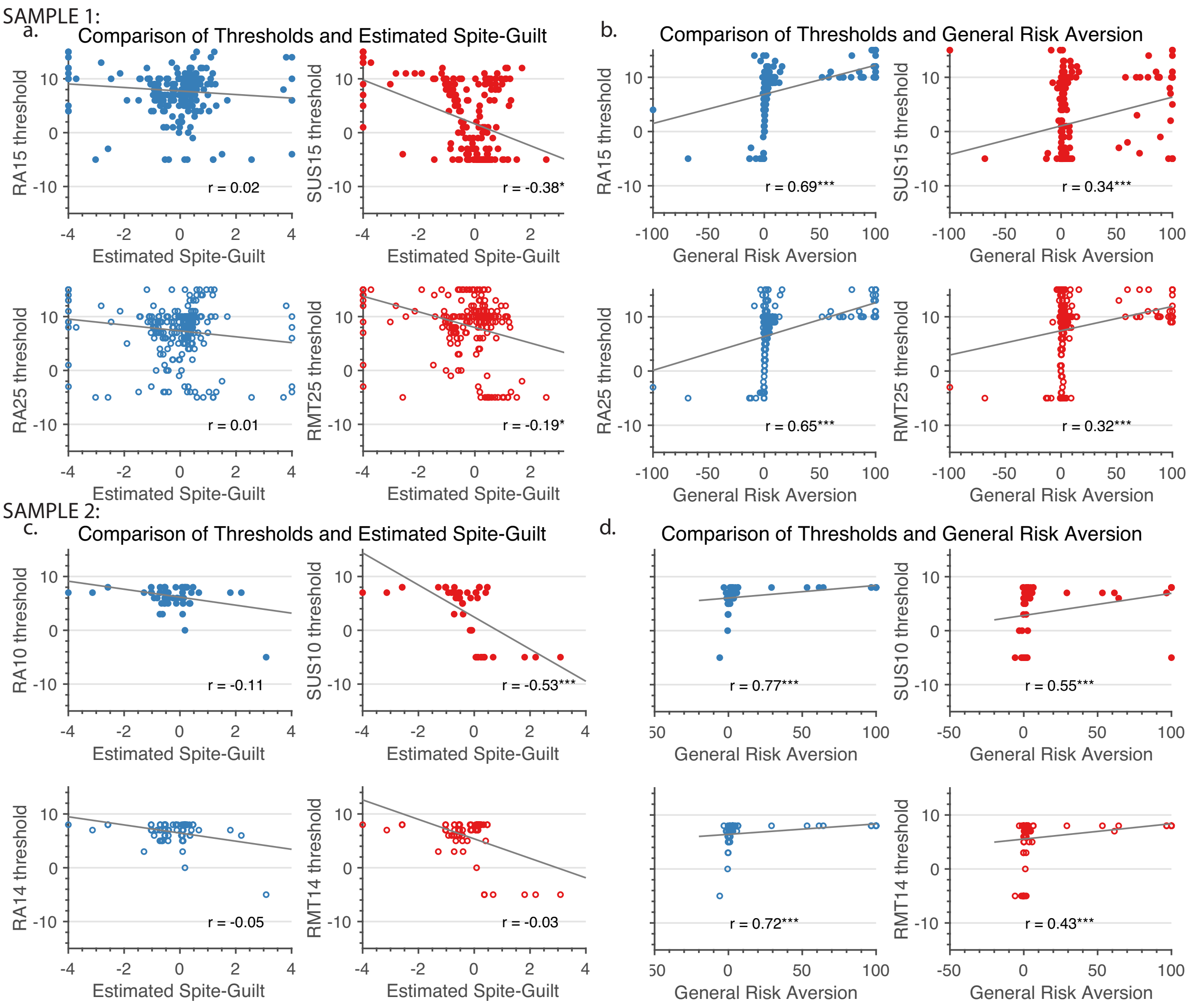

Figure S5. Estimated spite-guilt was most associated with the Suspiciousness condition in initial sample. *a) Estimated spite-guilt was most strongly correlated with the Suspiciousness condition thresholds (SUS15), and to a lesser degree was also associated with the Rational Mistrust condition thresholds (RMT25) b) Risk Aversion was highly correlated with all conditions.* The x-axis provides the given parameter estimate for each individual. The y-axis shows the estimated Heaviside threshold Ad value in which an individual switched from trusting to not trusting the partner. Lower threshold values represent more trust. *Blue represents trials against the coin partner, and red represents trials against the human partner. All correlations used the Spearman method. Gray lines indicate least squares regression line. p < .05*, p < .01**, p < .001****

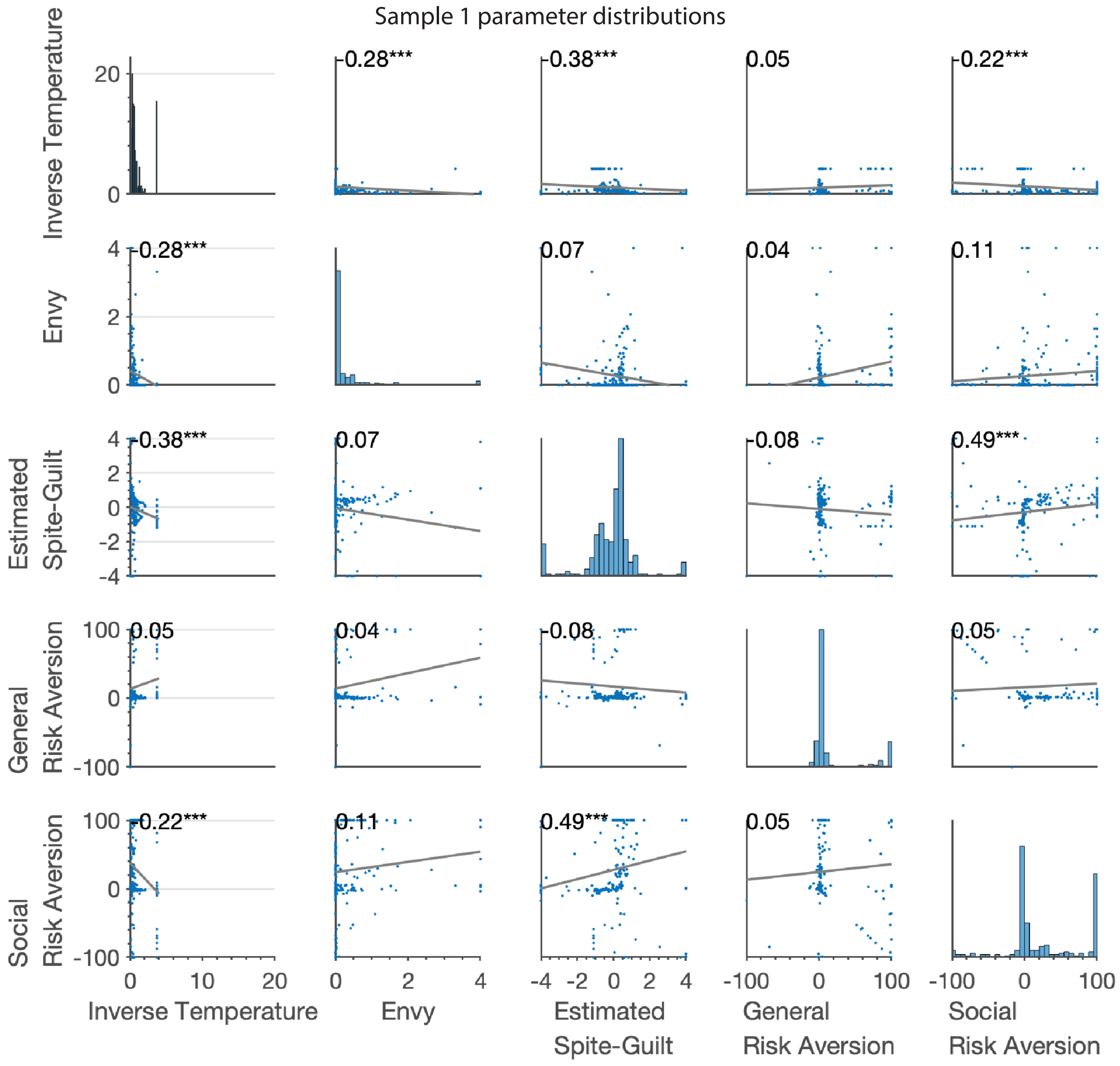

Figure S6. Parameter Correlations for Sample 1. *Correlation matrix of the five parameter distributions for Sample 1. Values in the upper left corner of each graph show the spearman correlation. Y-axis labels for all show the range for the parameter estimations. Gray lines indicate least squares regression line. $p < .05$*, $p < .01$**, $p < .001$****

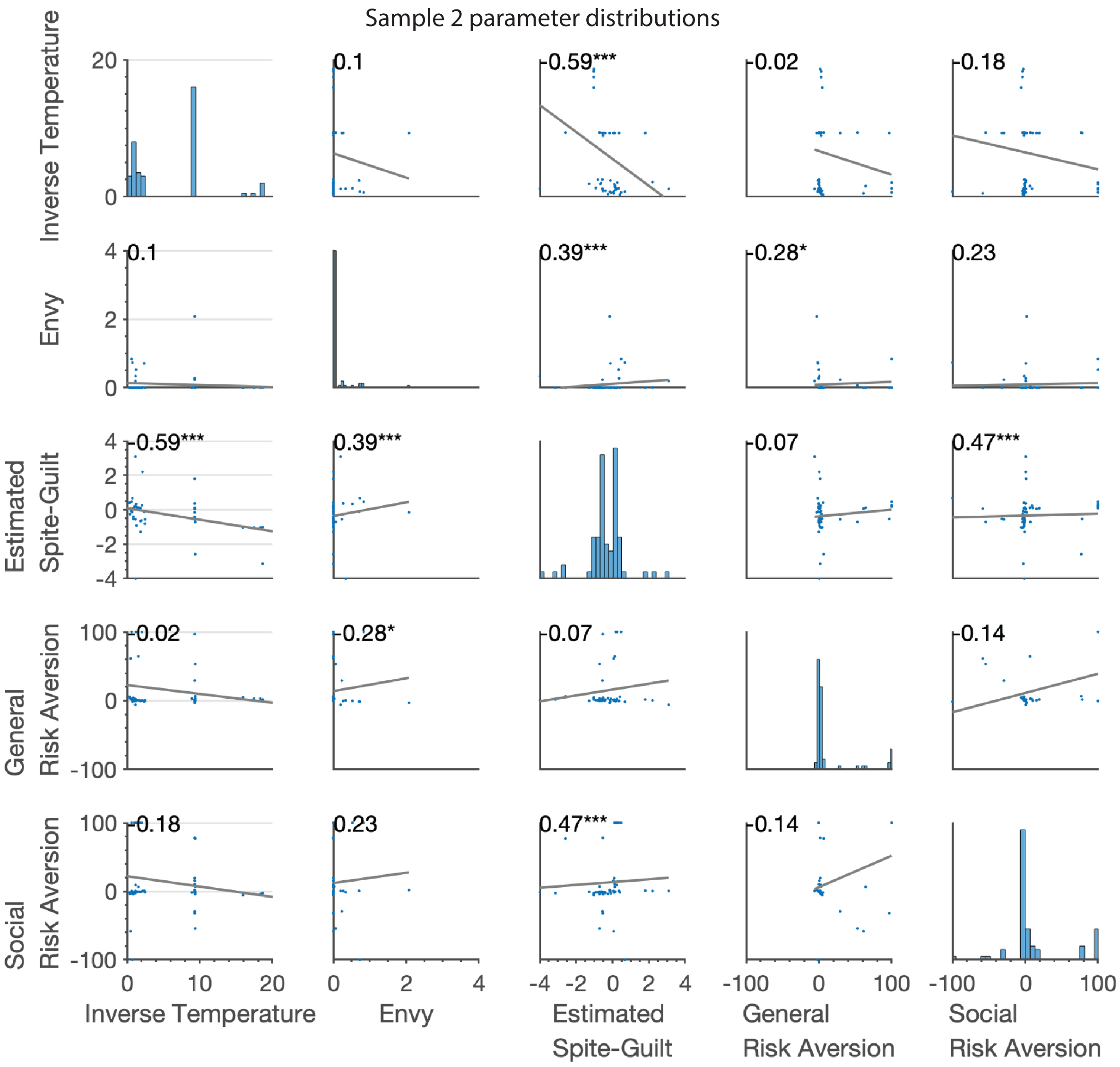

Figure S7. Parameter Correlations for Sample 2. *Correlation matrix of the five parameter distributions for Sample 2. Values in the upper left corner of each graph show the spearman correlation. Y-axis labels for all show the range for the parameter estimations. Gray lines indicate least squares regression line. p < .05*, p < .01**, p < .001****

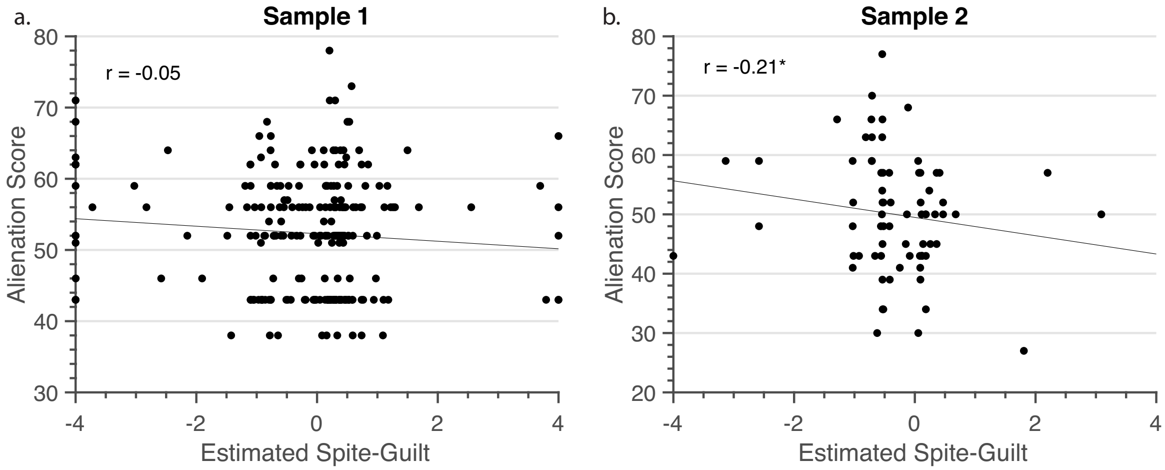

Figure S8. Correlations of MPQ-Alienation and Estimated Spite-Guilt for a) Sample 1 and b) Sample 2. $p < .05$*, $p < .01$**, $p < .001$***

Table S1

Descriptions of model comparisons.

Model	BIC Scores	# of parameters	Parameters
**Random**	28064	0	None
**Fehr-Schmidt**	20556	2	λ, α
**H2 Coin Vs. Human**	23140	4	λ_C, α_C, λ_H, α_H
**H2 Shared Inverse Temperature**	21427	3	λ, α_C, α_H
**H3 Coin vs. Human Risk Aversion**	20494	4	λ, α, R_C, R_H
**H4a Estimate Spite-Guilt: Human**	20025	4	λ, α_C, α_H, β'_H
**H4a Estimate Spite-Guilt: Coin**	22481	4	λ, α_C, α_H, β'_C
**H4a Estimate Spite-Guilt: Both**	20789	5	λ, α_C, α_H, β'_C, β'_H
**H4a Estimate Spite-Guilt: Human & Shared Risk Aversion**	20202	4	λ, α, β'_H, R
**H4a Estimate Spite-Guilt: Human & Split Risk Aversion**	21719	5	λ, α, β'_H, R_C, R_H
**H4b Estimate Spite & Guilt: Split Envy**	23455	5	λ, α_C, α_H, β'_H, S'_H
**H4b Estimate Spite & Guilt: Shared Envy**	24904	4	λ, α, β^*_H, S'_H
**H4b Estimated Spite & Guilt: Split Envy & Split Risk Aversion**	26252	7	λ, α_C, α_H, β'_H, S'_H, R_C, R_H
**H4b Estimated Spite & Guilt: Shared Envy & Split Risk Aversion**	24424	6	λ, α, β'_H, S'_H, R_C, R_H
**Final model Spite Sensitivity**	20855	5	λ, α, β'_H, R_G, R_S

Note. Bayesian Information Criterion (BIC) scores for a Random model, Fehr-Schmidt model, and Spite Sensitivity model for Sample 1. λ = noise/exploration, α = envy, β' = estimated spite-guilt, β^* = estimated guilt, R = Risk Aversion, R_G = General Risk Aversion, R_S = Social Risk Aversion, S' = estimated spite. Subscripts C or H denote parameters applied only to the coin or human partner, respectively.

Table S2

Model Results of Behavioral and Computational Measures.

Variable	Age	Education	Sex
**MPQ-Alienation**	Estimate = -.145, SE = .20, t = .458, p = .46	Estimate = -.248, SE =.54, t = 2.11, p = .647	**Estimate = 2.37, SE = 1.12, t = 2.11, p = .036***
**MPQ-Harm Avoidance**	Estimate = .18, SE = 1.08, t = .937, p = .349	Estimate = .459, SE = .519, t = .886, p =.377	**Estimate = 2.97, SE = 1.07, t = 2.76, p = .006****
**SUS15 threshold**	Estimate = .05, SE = .18, t = .29, p = .773	Estimate = .146, SE = .47,t = .31, p = .756	Estimate = .350, SE = .979, t = .36, p = .721
**RMT25 threshold**	Estimate = .054, SE = .135, t = .405, p = .685	Estimate = .385, SE = .364, t = 1.058, p = .29	**Estimate = 1.53, SE = .754, t = 2.03, p = .043***
**RA15 threshold**	Estimate = .058, SE = .097, t = .599, p = .549	Estimate = .15, SE = .26, t = .572, p = .567	**Estimate = 2.36, SE = .54, t = 4.34, p < .001*****
**RA25 threshold**	Estimate = .02, SE = .11, t = .20, p = .84	Estimate = .109, SE = .32, t = .339, p = .73	**Estimate = 2.78, SE = .66, t = 4.15, p < .001*****
**SM 15 threshold**	Estimate = .047, SE = .11, t = .41, p = .68	Estimate = .09, SE = .30, t = .29, p = .76	Estimate = .138, SE = .64, t = .21, p = .83
**SM 25 threshold**	Estimate = .23, SE = .19, t = 1.17, p = .24	Estimate = .23, SE = .53, t = .43, p = .66	Estimate = 1.12, SE = 1.12, t = 1.00, p = .317
**Lambda**	Estimate = .003, SE = .029, t = .132, p = .894	Estimate = .079, SE = .078, t = 1.01, p = .310	**Estimate = .329, SE = .163, t = 2.02, p = .044***
**Envy**	Estimate = .01, SE = .01, t = .55, p = .576	Estimate = .049, SE = .049, t = .988, p = .323	Estimate = .067, SE = .102, t = .651, p = .515
**Estimate Spite-Guilt**	Estimate = .017, SE = .03, t = .50, p = .616	Estimate = .081, SE = .095, t = .855, p = .393	Estimate = .106, SE = .197, t = .539, p = .589
**General Risk Aversion**	Estimate = .11, SE = .898, t = .12, p = .902	Estimate = 2.369, SE = 2.42, t = .97, p = .329	**Estimate = 14.0, SE = 5.02, t = 2.79, p = .005****
**Social Risk Aversion**	Estimate = 1.16, SE = 1.34 t = .867 , p = .38	Estimate = 6.79, SE = 3.62, t = 1.87, p = .062	Estimate = 9.36, SE = 7.52, t = 1.24, p = .21

Supplemental Methods & Results

Questionnaires

In addition to the Multidimensional Personality Questionnaire Brief Form (MPQ-BF)(Patrick et al., 2002), each participant completed a computerized personality inventory comprised of items from the Schizotypal Personality Questionnaire – Brief Form (SPQ-B) (Raine & Benishay, 1995), the Interpersonal Trust Scale (Rotter, 1967), and the Trust-Suspicion Scale (Heretick, 1981). These scales were chosen because they were broadly available, frequently used within their own domains and had a similar response format. Items were randomized throughout the questionnaire and scored on a 4-point Likert scale (1 = always true, 2 = mostly true, 3 = mostly false, 4 = always false). We chose to focus on the MPQ-Alienation subscale as it is well-studied and was consistent with previous work in this area (Johnson et al., 2009).

Analyses

The formulas for the regression models included:

1. `Behavioral results: Choice~t*ad*DA+(1|subID)`
2. `Alienation comparison: Choice~t*ad*DA*MPQAL+(1|subID)`
3. `Variables of interest: (variable)~Age+Edu+Sex`

Where t is the temptation, ad is the adverse payoff, DA is the decision agent, and subID accounts for each individual using random effects. Variables of interest included threshold values for each condition, MPQ-Alienation, and the model parameters. These results can be found in Table S2.

Hypothesis testing

The hypotheses stated in the introduction were supported. In brief, we showed that the Fehr-Schmidt model outperformed a random model yet was not an adequate representation of behavior seen in the paradigm (H1). We tested H2, that modeling the decision agent separately would improve the model and showed that multiple envy parameters were not better than a single envy parameter seen in the Fehr-Schmidt model. However, in our final model, we identified two instances in which separating by decision agent improved the model; estimated spite-guilt (modeled as an anticipation of the partner's guilt) was only applied to the human partner, and we had two separate risk aversion parameters for overall risk and risk for just the human partner interactions. Our next hypothesis was that risk aversion would improve the model (H3). We found that separating the risk aversion parameters by decision agent improved the Fehr-Schmidt model yet did not simulate the expected pattern of behavior. The final model

included two separate risk aversion parameters which related to distinct behavior in the game. We found that the additional estimated spite-guilt parameter was important to model suspicious behavior, thus allowing us to model spite sensitivity for the first time (H4a). However, separating positive and negative guilt into guilt and spite did not improve the model (H4b). Our results suggest that a fear of spite and risk aversion are separate mechanisms in decision-making.

Hypothesis 1

We tested H1 by comparing the Fehr-Schmidt model to the random model (Figure S1). Based on BIC scores, the Fehr-Schmidt model (Figure 2) is better than a random model (Table S1). Just adding a single parameter of envy α_1 adds significant value to the performance of the model. However, when looking at the graph of actual to simulated data, we see that the Fehr-Schmidt model does not represent the decisions in the Suspiciousness condition well (Figure 2).

Hypothesis 2

To test H2, we assumed that the addition of the coin will act as a random partner (i.e., probability is equal to .5), whereas the human partner will behave in a more rational and predictable way. To test this, we first separated out the two conditions using the Fehr-Schmidt model, such that each condition had its own envy α and inverse temperature λ. However, we also tested models in which there is only one λ shared between the two conditions, even though there are two envy parameters (H2 Coin vs. Human in Table S1). Finally, we also tested if envy was only necessary for one of the conditions (either coin or partner), but not the other. We hypothesize that the coin, as a random entity (50-50), will not require an envy parameter, but the human partner will. In addition, there will only be a shared λ across the two conditions.

To compare the two partner conditions, we also tested if multiple envy α and inverse temperature λ parameters were necessary. Of these sets of models, we found that the best model included separate envy α parameters for each condition, but a shared λ across each softmax equation for the player and the estimated partner's behavior (H2 Shared Inverse Temperature in Table S1). However, modeling multiple envy parameters was not significantly better than a single envy parameter for both in the Fehr-Schmidt model.

Hypothesis 3

H3 examined the impact of risk aversion. The Risk Aversion conditions with the coin partner assisted in measuring risk aversion of the individual, since the coin is essentially a random partner. We model risk aversion (R_1) by comparing the monetary amount of the adverse payoff x_1 to the amount of the safe option, S.

$$U_{ADVERSE}(x) = x_1 - \alpha_1\, max\{x_2 - x_1, 0\} - R_1 max\{S - x_1, 0\}; \text{ Risk Aversion}$$

We modeled risk aversion alone and with the estimated partner's guilt above. We also model risk aversion for both decision agents, human partner alone, and coin alone.

Several models were tested, including separating risk aversion between the two different conditions. The addition of risk aversion was most effective when envy α_1 and inverse temperature λ were shared across conditions, but risk aversion was considered separately (H3 Coin vs. Human Risk Aversion in Table S1). Despite the improved BIC score, the simulated data did not strongly separate the two human conditions (Rational Mistrust and Suspiciousness).

Hypothesis 4

Estimated Spite-Guilt

We tested H4 by modeling the partner's behavior in several different ways. Because it is likely that the second mover is not deciding randomly, we added a parameter in which the first mover estimates the probability of the second mover's choice. We call this parameter estimated spite-guilt, as it is the ability to estimate the partner's guilt. We hypothesize this parameter will improve the model above and beyond the Fehr-Schmidt model of just envy α_1. We model this by estimating the parameter of the partner's guilt (β_2). Additionally, a human partner may also include some risk aversion associated with the potential loss of money, as well as require an estimate of the amount of guilt of the partner. Because in our dataset there is never a time in which the first mover has more money than the second mover, we simplify the equation to remove the partner's envy α_2.

$$U_{TEMPTATION}(x) = x_2 - \beta_2 \, max\{x_2 - x_1, 0\}; \text{ Estimated Spite-Guilt}$$

From this equation, we again use the softmax equation to estimate the probability of the second mover choosing the temptation. The probability is used as *p*, which influences weighted utilities of the two possible outcomes should the first mover choose to trust.

$$probability(Temptation) = \frac{e^{\lambda * U_{TEMPATION}}}{e^{\lambda * U_{MUTUAL}} + e^{\lambda * U_{TEMPTATION}}}$$

We tested the model with three possible inverse temperature λ options — a shared λ across both individuals, distinct λ for each player, and no estimated λ for the second mover. As we assume that the second mover will have guilt, but not the coin, we additionally model the estimated partner's guilt β_2 in three separate ways: both the fair coin and human partner, just the human, and just the coin (as a sanity check). When partner spite-guilt was not estimated, *p* was assumed to be .5.

We compared several models using estimated spite-guilt, including separating out the behavior between coin and human partners. First, we tested estimated spite-guilt with envy but without risk aversion. Estimated spite-guilt was most effective in the model when it was only used to estimate the human partner's behavior (H4a Estimated Spite-Guilt: Human), compared to estimating both partner conditions separately (H4a Estimated Spite-Guilt: Both) or just for the

coin partner (H4a Estimated Spite-Guilt: Coin). The addition of estimated spite-guilt for only the human partner improved the model compared to the Fehr-Schmidt model.

When including risk aversion, we compared two models of a human partner's guilt with either a single shared envy and a shared risk (H4a Estimated Spite-Guilt: Human & shared Risk Aversion) or shared envy with a separated risk (H4a Estimated Spite-Guilt: Human & Split Risk Aversion). While the second model of risk aversion and estimated spite-guilt was a higher BIC, the simulated data using the H4a Estimated Spite-Guilt: Human & Split Risk Aversion model matched the behavior better. We also compared these models to splitting Risk Aversion as a general term (for all conditions and decision agents – General Risk Aversion) and one only for the human partner (Social Risk Aversion). This model had improved BIC scores and simulated data matched the expected pattern of behavior (Spite Sensitivity model in Table S1).

Estimated Spite & Guilt Separately

H4b was tested by separating the estimated spite-guilt into negative guilt (spite) and positive guilt. Instead of allowing guilt to be negative or positive, we modeled spite separately as a variable adding positive utility to the partner's advantage over the first mover. Each parameter will be constrained to positive values only. This will test if there is a bivalent relationship between guilt and spite, as opposed to a continuum with negative or positive guilt.

$$U_{TEMPTATION}(x) = x_2 - \beta_2\, max\{x_2 - x_1, 0\} + S_2\, max\{x_2 - x_1, 0\}$$; Partner Spite

Including two envy parameters with estimated guilt and spite for the human partner only (H4b Estimated Spite & Guilt: Split Envy) was better than using one envy parameter with estimated guilt and spite (H4b Estimated Spite & Guilt: Shared Envy).

However, when we additionally included risk aversion, we found that 1 envy and 2 risk parameters (along with partner spite and guilt) were better fit (H4b Estimated Spite & Guilt: Shared Envy & Split Risk Aversion) than 2 envy and 2 risk parameters (H4b Estimated Spite & Guilt: Split Envy & Split Risk Aversion).

Based on BIC and simulated graphs, we identified that a single envy parameter, two risk aversion parameters, and human partner estimated guilt (as a continuous negative or positive value) best fit the data (Spite Sensitivity in Table S1).

Social Risk Aversion

To further examine this relationship between social risk aversion and the human partner thresholds, we compared the difference between the two conditions, RMT25-SUS15, and identified a negative relationship ($r_s(241) = .288$, $p < .001$). This relationship means that when RMT25 thresholds are higher than SUS15, social risk aversion is also higher such that more risk aversion in the Rational Mistrust condition drove the social risk aversion parameter estimation.

As we had hoped it would represent general mistrust of other partners, it appears to match our expectations; however, the negative relationship with SUS15 thresholds seems to be driven by a disconnect between the SUS15 condition and the RMT25 condition. There was also a moderately strong correlation between the estimated spite-guilt parameter and the social risk aversion parameter ($r_s(241) = .490$, $p < .001$). Social risk aversion was not correlated with the lower Risk Aversion condition thresholds (RA15: $r_s(241) = .10$, $p = .139$) and somewhat associated with the higher Risk Aversion condition thresholds (RA25: $r_s(241) = .13$, $p = .046$). As the social risk aversion parameter was added to the general risk aversion parameter in the human partner conditions, it is necessarily more associated with the human partner conditions.

Variables and Demographic Predictors

We examined the extent to which demographic variables predicted behavioral and computational measures in our study. See Table S2 for details. Overall, sex differences predicted several measures in the study. Men were more alienated and less harm avoidant in the MPQ measures; Previous research has shown that men are more likely to have higher alienation scores but no differences in harm avoidance (Finkel & McGue, 1997). In terms of behavior, men had lower thresholds in the RA15, RA25, and RMT25 conditions, suggesting men were less risk averse than women. Finally, computational variables corroborated this result, in which men had lower general risk aversion and higher inverse temperature. These results are consistent with previous literature on the Trust Game, in which men were more trusting of unknown partners (Lemmers-Jansen et al., 2017). Women have also been shown to be more risk averse in financial decision making (Charness & Gneezy, 2012). Overall, this suggests that women in our sample are more risk averse in their behavior, personality measures, and computational measures than their male counterparts.

Second Mover Analyses

We compared thresholds in the First Mover Game to those from the Second Mover Game and found that they were significantly negatively correlated (SUS15: $r_s(241) = -.15$, $p <.001$; RMT25: $r_s(241) = -.29$, $p <.001$), in which higher distrust (and higher thresholds) in the First Mover Game would be associated with Higher selfishness and spite (lower thresholds) in the Second Mover Game and vice versa. This result suggests that players assumed their partners were like themselves (Figure S1). We additionally examined the relationship between Second Mover thresholds and MPQ-Alienation, as previous work has suggested that paranoia may influence generosity (Raihani et al., 2020). However, we did not see a correlation between thresholds in either sample for either condition of the Second Mover Game and MPQ-Alienation (p's >.46).

References

Charness, G., & Gneezy, U. (2012). Strong Evidence for Gender Differences in Risk Taking. *Journal of Economic Behavior and Organization*, *83*(1), 50–58. https://doi.org/10.1016/j.jebo.2011.06.007

Finkel, D., & McGue, M. (1997). Sex differences and nonadditivity in heritability of the multidimensional personality questionnaire scales. *Journal of Personality and Social Psychology*, *72*(4), 929–938. https://doi.org/10.1037/0022-3514.72.4.929

Heretick, D. M. L. (1981). Gender-Specific Relationships Between Trust-Suspicion, Locus of Control, and Psychological Distress. *The Journal of Psychology*, *108*(2), 267–274. https://doi.org/https://doi.org/10.1080/00223980.1981.9915274

Johnson, M. K., Rustichini, A., & MacDonald III, A. W. (2009). Suspicious personality predicts behavior on a social decision-making task. *Personality and Individual Differences*, *47*(1), 30–35. https://doi.org/10.1016/j.paid.2009.01.050

Lemmers-Jansen, I. L. J., Krabbendam, L., Veltman, D. J., & Fett, A. K. J. (2017). Boys vs. girls: Gender differences in the neural development of trust and reciprocity depend on social context. *Developmental Cognitive Neuroscience*, *25*, 235–245. https://doi.org/10.1016/j.dcn.2017.02.001

Patrick, C. J., Curtin, J. J., & Tellegen, A. (2002). Development and validation of a brief form of the Multidimensional Personality Questionnaire. *Psychological Assessment*, *14*(2), 150–163.

Raihani, N., Martinez-Gatell, D., Bell, V., & Foulkes, L. (2020). Social reward, punishment, and prosociality in paranoia. *Journal of Abnormal Psychology*, *130*(2), 177–185. https://doi.org/10.1037/abn0000647

Raine, A., & Benishay, D. (1995). The SPQ-B: A Brief Screening Instrument for Schizotypal Personality Disorder. *Journal of Personality Disorders*, *9*(4), 346–355.

Rotter, J. B. (1967). A new scale for the measurement of interpersonal trust. *Journal of Personality*, *35*(4), 651–655. https://doi.org/https://doi.org/10.1111/j.1467-6494.1967.tb01454.x
